# 3D models of the hematopoietic stem cell niche under steady-state and active conditions

**DOI:** 10.1038/s41598-017-04808-0

**Published:** 2017-07-04

**Authors:** Lisa Rödling, Ivo Schwedhelm, Saskia Kraus, Karen Bieback, Jan Hansmann, Cornelia Lee-Thedieck

**Affiliations:** 1 Karlsruhe Institute of Technology (KIT), Institute of Functional Interfaces, Hermann-von-Helmholtz-Platz 1, 76344 Eggenstein-Leopoldshafen, Germany; 2 Institute of Transfusion Medicine and Immunology Mannheim, Medical Faculty Mannheim, Heidelberg University; German Red Cross Blood Donor Service Baden-Württemberg-Hessen, 68167 Mannheim, Germany; 3 Institute for Tissue Engineering and Regenerative Medicine, University of Würzburg, 97070 Würzburg, Germany

## Abstract

Hematopoietic stem cells (HSCs) in the bone marrow are able to differentiate into all types of blood cells and supply the organism each day with billions of fresh cells. They are applied to cure hematological diseases such as leukemia. The clinical need for HSCs is high and there is a demand for being able to control and multiply HSCs *in vitro*. The hematopoietic system is highly proliferative and thus sensitive to anti-proliferative drugs such as chemotherapeutics. For many of these drugs suppression of the hematopoietic system is the dose-limiting toxicity. Therefore, biomimetic 3D models of the HSC niche that allow to control HSC behavior *in vitro* and to test drugs in a human setting are relevant for the clinics and pharmacology. Here, we describe a perfused 3D bone marrow analog that allows mimicking the HSC niche under steady-state and activated conditions that favor either HSC maintenance or differentiation, respectively, and allows for drug testing.

## Introduction

Hematopoietic stem cells (HSCs) are the stem cells of the blood and master the continuous production of new blood cells throughout life^1–3^. Due to their ability to reconstitute the entire cellular compartment of the blood, HSCs are routinely transplanted to treat patients with life-threatening hematological disorders such as leukemia. Upon transplantation of healthy HSCs, isolated from the bone marrow or peripheral blood of a matching donor, the cells can engraft in the patient’s bone marrow and reconstitute healthy hematopoiesis^4^.

The clinical application of HSCs is limited by the fact that the number of patients in need exceeds the number of matching donors. One approach to overcome this gap in supply is the use of HSCs from umbilical cord blood (UCB)^5, 6^. For promising engraftment and fast hematopoietic recovery, a minimal cell dose of 2.5 × 10^7^ cells per kilogram bodyweight is required^7^. The dose of stem cells in one cord blood unit is often too small for successful reconstitution of the hematopoietic system. *Ex vivo* expansion of HSCs from UCB is therefore an elegant approach to circumvent the shortage of available HSCs^8^. The current clinical strategy to increase the number of cells is to transplant two partially human leukocyte antigen (HLA)-matched UCB units^7^. In order to minimize the risk for the transplanted patients, a similar strategy is used when applying *ex vivo* expanded HSC in clinical trials: one unmanipulated unit containing long-term repopulating HSCs is transplanted together with hematopoietic (stem) cells that were expanded *in vitro* from a second unit. Strategies for *ex vivo* expansion of HSCs that have been tested in clinical trials phase I/II comprise co-culture with mesenchymal stem/stroma cells (MSCs)^9^, stimulation of the notch-receptor^10^ and cultivation in the presence of the copper chelator tetraethylenepentamine (StemEx)^11, 12^, the small molecule nicotinamide^13, 14^ or the aryl hydrocarbon receptor antagonist StemRegenin 1 (SR1)^15, 16^.

The challenge of successful *ex vivo* expansion of HSCs is that the cells need to proliferate whilst preserving their stem cell properties: the ability to differentiate into all blood cell lineages and to undergo self-renewing cell divisions. Typically when cultured *ex vivo/in vitro*, HSCs quickly initiate differentiation and lose their stem cell properties when starting to proliferate^17^. Only *in vivo* in their natural environment HSCs can proliferate and maintain their stem cell phenotype at the same time. This is ensured by a specialized microenvironment in the bone marrow: the stem cell niche^18^. The concept of a HSC niche which regulates HSC behavior was first published by Schofield in 1978, who also coined the term “stem cell niche”^19^. These niches harbor a variety of different factors that all—individually and in concert—influence HSC behavior. In the niche, HSCs are in close vicinity of supporting niche cells including osteoblasts and MSCs^20–22^. Further signals derive from the extracellular matrix and also the three-dimensional (3D) architecture of the niche impacts HSCs^23–29^. Artificial reconstruction of all of these niche components in one biomaterial is a current approach to simulate the *in vivo* situation of HSCs with the goal to control stem cell behavior *in vitro* ^30^. Additionally to these rather solid components of the niche, soluble factors are crucial in regulating HSCs. Cytokines and hormones for example ensure cell–cell signaling across longer distances as well as on the short range in a paracrine or autocrine fashion. In this way soluble factors have a tremendous effect on HSC behavior and fate^31, 32^. Furthermore, the reduced availability of oxygen due to hypoxia in the bone marrow as well as the availability of specific nutrients are critical for the niche to function^33–35^. The flow of blood and liquid inside of the niche applies a certain shear stress and mechanical forces^36^. All of these factors have been shown to influence HSCs and explain the large differences observed in their behavior *in vivo* in their niche—where maintenance and differentiation are balanced and tightly regulated—and *in vitro* in state-of-the-art 2D cell culture—where the self-renewing potential is quickly lost in favor of differentiation^17^. Therefore, standard cell culture is not sufficient to mimic the *in vivo* situation of HSCs—neither for targeted proliferation or differentiation of HSCs, nor for assessing the efficacy or toxicity of drugs on the hematopoietic compartment of the bone marrow.

To overcome the limitations of 2D cell culture, approaches including sophisticated biomaterials or bioreactors are often applied to mimic the natural situation of HSCs more closely. The applied biomaterials can be roughly subdivided according to the used materials and their architecture. Besides some inorganic biomaterials such as hydroxyapatite^37^, mostly hydrogels are used to mimic the HSC niche. These hydrogels are produced from natural (e.g. heparin, matrigel, collagen, silk) or synthetic polymers (including polyethylene glycol (PEG) or polyacrylates). The architecture of the hydrogels that were applied to culture HSCs differs strongly and ranges from flat gel pads via microwell substrates as well as fibrous or porous scaffolds to cell-encapsulating gels^27–29, 38–50^.

Multiple different bioreactor setups have been used to improve HSC culture. Cultures in rotating wall vessel bioreactors and orbital shake flasks with intermittent shaking both resulted in an elevated multiplication of cells expressing the surface marker CD34^+^—that labels hematopoietic stem and progenitor cells (HSPCs)—compared to static cultures^51, 52^. Studies on more complex dynamic 3D setups including a co-culture of lineage-negative UCB cells with bone marrow stroma cells in a hollow fibre continuous perfusion reactor, however, reported expansion rates similar to static 2D co-culture setups^53^ or an enrichment of myeloid progenitors rather than of HSCs^54^. The bone marrow-on-a-chip by Torisawa *et al*.^55^—despite not being fully synthetic—was probably one of the most advanced bone marrow analogs described so far.

Myelosuppression (so called bone marrow suppression) is one of the most common toxic side effects of anti-proliferative chemotherapeutics and a common cause of death of treated cancer patients^56^. Many chemotherapeutic agents target quickly dividing cells such as tumor cells, but at the same time they also attack organs with a high turnover such as the hematopoietic compartment that produces billions of cells each day^57^. Myelosuppression describes a decrease of blood cell production (hematopoiesis) in the bone marrow which yields a reduction in blood cells, including immune cells (leukocytes), red blood cells (erythrocytes) and platelets (thrombocytes) that can lead to life-threatening conditions due to the susceptibility to infections, anemia and bleeding^58^. Accurate prediction of the myelotoxic risk by 3D *in vitro* models of the human bone marrow could increase the success rate and speed of the development of novel chemotherapeutics.

To date, most studies that aimed at developing *in vitro* models of the bone marrow relied on standard cell culture of cell lines or primary cells and did not take the physical parameters of the HSC niche into account. Other studies concentrated either on the impact of one or more material parameters (e.g., topography, stiffness or 3D architecture) or on the influence of soluble signals. There is only limited data on how the ‘solid’ and the ‘liquid’ compartment act in concert in biomaterial-based approaches for mimicking the HSC niche. Therefore, one aim of the current study was to investigate how fluid flow inside of a multi-parameter biomaterial influences HSPC proliferation, differentiation and maintenance. As multi-parameter biomaterial we took advantage of a 3D artificial bone marrow analog^28^ that mimics crucial aspects of the natural HSC niche (Supplementary Figure S1). It consists of a macroporous PEG hydrogel that resembles the macroporous 3D architecture of trabecular bone—the place where the red bone marrow and thus HSC niches are located. To recapitulate the adhesive properties of the extracellular matrix the hydrogel is equipped with RGD-peptides to which cells can adhere via integrin receptors. RGD is a minimal adhesive peptide sequence found in extracellular matrix molecules such as fibronectin. Support by supporting niche cells found naturally in the neighborhood of HSCs in the niche, is mimicked by co-culture with MSCs. This bone marrow analog was shown to be suitable for HSPC culture and to enhance the HSPC expansion in comparison to conventional 2D cell culture^28^. In the present study this biomaterial was advanced by integration into a bioreactor allowing perfusion of the biomaterial with medium while culturing HSPCs. Thereby, nutrients, oxygen and secreted cytokines are actively transported with the medium through the bone marrow analog while in a static culture the transport of these factors is limited to diffusion. This system of static versus dynamic culture was used to study how perfusion and the associated processes affect HSPC behavior in a simplified artificial HSC niche regarding proliferation, differentiation and maintenance of stemness. The aim of these studies was to develop an *in vitro* model that mimics the human stem cell niche either under steady-state conditions which favor stem cell maintenance or in an activated state as it occurs in alarm situations such as blood loss or infections, which fosters blood cell production by differentiation. Such systems are promising (i) for the design of bioreactors for HSC expansion or differentiation, and (ii) as *in vitro* test platform for myelosuppressive drugs.

## Results

### Dynamic HSPC culture with perfusion in a 3D bone marrow analog

**Figure 1:**
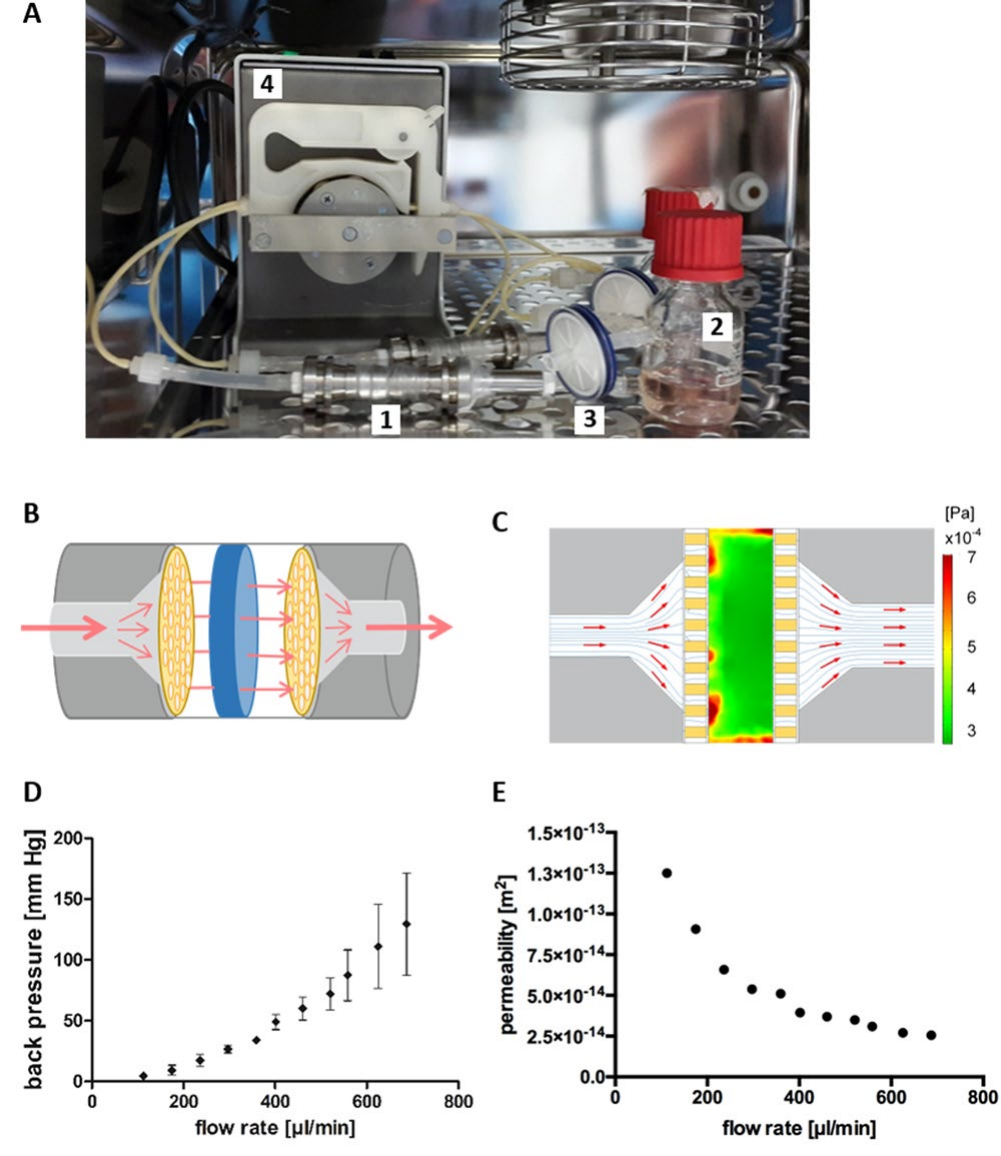
Perfusion of macroporous scaffolds in the bioreactor. (**A**) Experimental setup of perfusion reactors harboring macroporous scaffolds inside an incubator. 1: perfusion reactor; 2: medium reservoir; 3: ventilation filter; 4: peristaltic pump. (**B**) Schematic drawing of the macroporous hydrogel within the bioreactor. The medium is led unidirectional (red arrow) via perforated plates (yellow) through the macroporous hydrogel (blue). (**C**) Computational fluid dynamics of perfused bioreactor (sectional view). The perforated plate is depicted in yellow, flow direction is shown by red arrows and stream lines (blue), and flow-derived shear stress is indicated by color range. (**D**) The back pressure (depicted on the y-axis) in front of the macroporous hydrogel was monitored in dependency of the flow rate (x-axis). N = 3 independent experiments. (**E**) The computational fluid flow model was harnessed to extract data on hydrogel permeability at increasing flow rates. Permeability is shown on the y-axis and flow rates on the x-axis.

A macroporous hydrogel that mimics the spongy architecture of the bone marrow containing cancellous bone was applied as scaffolding material in the bone marrow analog. The hydrogel scaffold was colonized with HSPCs and MSCs for 16 hours and inserted into a perfusion reactor. The perfusion reactor was connected via tubing to a medium reservoir bottle from which medium was continuously pumped through the reactor (Fig. 1A,B).

To characterize the assembled perfusion setup, the back pressure resulting from the reactor with the macroporous hydrogel was monitored at stepwise increasing flow rates. The detected back pressure increased with increasing flow rates from 4.5 ± 0.5 mmHg to 130 ± 34 mmHg (mean ± standard deviation) (Fig. 1D). At flow rates higher than 687 µl/min (130 mmHg) the back pressure increased despite a constant flow rate, indicating that the hydrogel had collapsed and clogged the outlet of the reactor hindering the medium to flow through. Based on these back pressure measurements, the permeability of the hydrogel was calculated using computational fluid dynamics. Here, a non-linear decline of gel permeability ranging from 1.3 × 10^−13^ to 2.5 × 10^−14^ m² at increasing flow rates was obtained (Fig. 1E). Further, shear stress maxima of 7 × 10^−4^ Pa were detected on the outer boundaries of the scaffold (Fig. 1C). The remaining volume of the hydrogel showed homogenous shear distribution of around 3.5 × 10^−4^ Pa. The applicability of the perfusion reactor for HSPC/MSC co-cultures was tested at flow rates of 161, 115 and 69 µl/min. After 5 days of dynamic culture, only low numbers of cells were isolated from the hydrogels operated at high and intermediate flow rates. Only at the lowest flow rate substantial amounts of cells could be found inside of the hydrogel in the reactor.

To test the cell-compatibility of the perfusion bioreactor, the viability of the cells was assessed after 5, 9, 14 and 21 days of static and dynamic culture in the macroporous hydrogel. For a culture period of up to 9 days, more than 90% of the cells were viable under both culture conditions. The fraction of viable cells remained over 90% for up to 21 days in the static culture and dropped to 79% in the dynamic culture (Supplementary Figure S2). This decrease, however, was not significant in comparison to the static culture.

### Number of cells expressing the stem/progenitor cell marker CD34 upon static and dynamic culture

**Figure 2:**
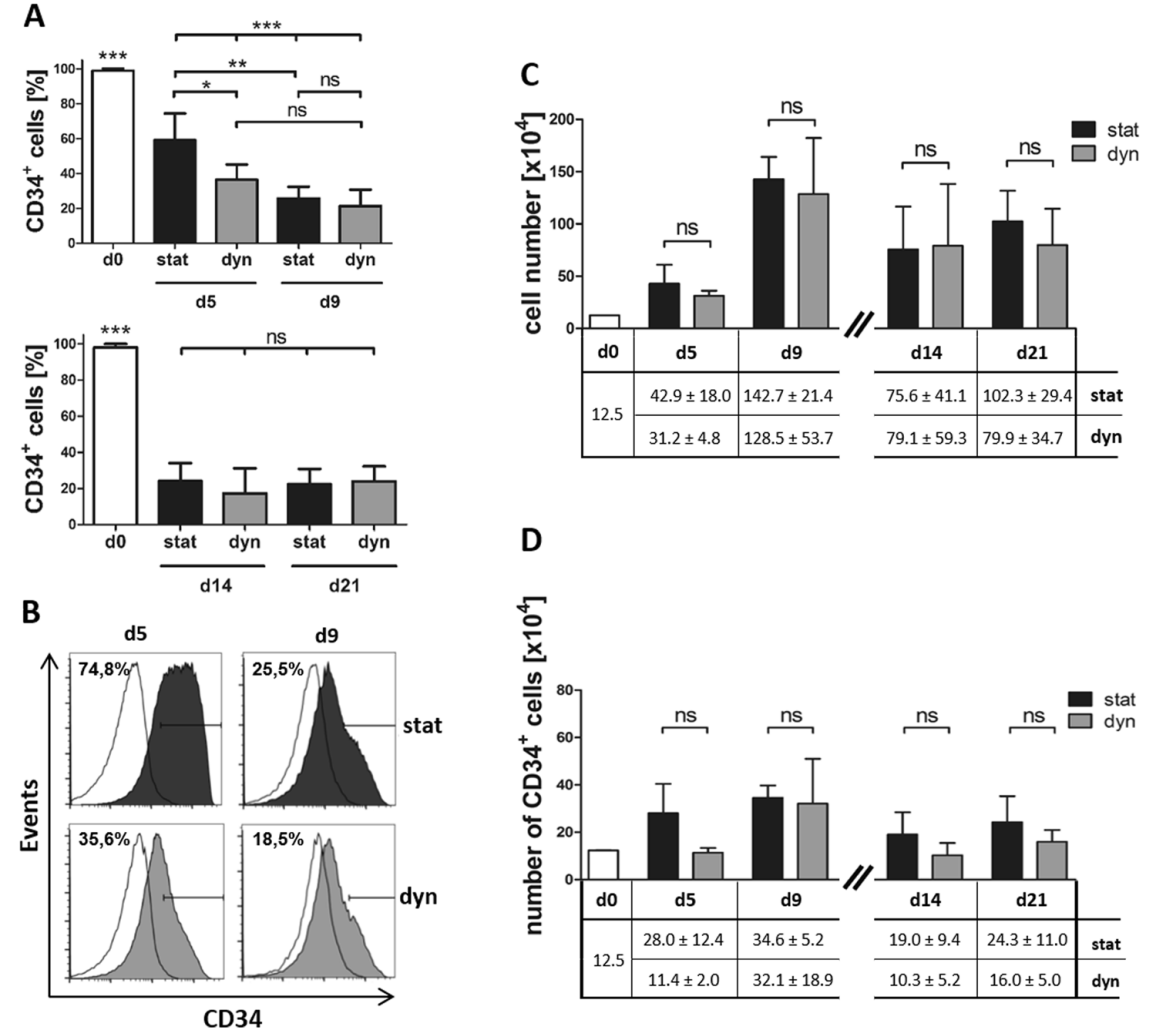
CD34 maintenance but not cell proliferation is influenced by the mode of culture. (**A**) Comparison of the proportion of CD34^+^ cells after static and dynamic culture over time. HSPCs were cultured under static or dynamic conditions and the percentage of CD34^+^ cells obtained on day 5 and day 9 or day 14 and day 21 was assessed by flow cytometry. Culture mode and day of analysis are given at the bottom. (**B**) Representative flow cytometry plots of CD34 expression analyses. Day of analysis is given on top, culture mode on the right. White histogram: isotype control; filled histogram: CD34 stained cells. Horizontal line marks gate for CD34^+^ cells. Percentages CD34^+^ cells are indicated at the top left of the respective histograms. (**C**) Total cell numbers retrieved from the scaffolds after static or dynamic culture on the given day of culture. The table at the bottom gives exact cell numbers ± standard error of the mean (SEM). (**D**) Numbers of CD34^+^ cells obtained after static or dynamic culture on the given culture day based on the cell numbers and the measured percentages of CD34^+^ cells. Table at the bottom gives exact CD34^+^ cell numbers ± SEM. For d0, d5 and d9: N = 4; for d14 and d21: N = 3 independent experiments. Stat = static culture, dyn = dynamic culture.

To study the impact of perfusion on cell growth inside of the 3D scaffolds, the total number of cells that could be harvested after 5, 9, 14 and 21 days was determined. The number of cells increased from day 0 and peaked on day 9. Thereafter, the numbers of harvestable cells stagnated or decreased. The culture mode—static or dynamic—had no significant impact on the number of harvested cells at all time points (Fig. 2C). As HSPCs do not only proliferate but also differentiate during culture, the harvested cell populations were analyzed in more detail for the expression of the stem cell marker CD34, which is lost upon hematopoietic differentiation.

Compared to freshly isolated HSPCs (day 0), the fraction of CD34^+^ cells was significantly reduced already after 5 days in static as well as dynamic culture (Fig. 2A,B). In the dynamic culture the proportion of CD34^+^ cells dropped to 38% already after 5 days and levelled at ~20% after 9 days, which were maintained until day 21. In the static culture, the percentage of CD34^+^ cells was significantly higher than in the dynamic culture after 5 days. From day 9 forward, the fraction of CD34^+^ cells in the static culture diminished to levels similar to the ones found in the dynamic culture (Fig. 2A and B). Therefore, it appeared that during the first 5 days of culture in the 3D scaffolds the maintenance of CD34 expression was higher under static than under dynamic culture conditions. However, when calculating the number of harvested CD34^+^ cells (by multiplying the total cell number by the percentage of CD34^+^ cells), no significant differences were detectable between the static and dynamic culture at the individual time points (Fig. 2D).

**Figure 3:**
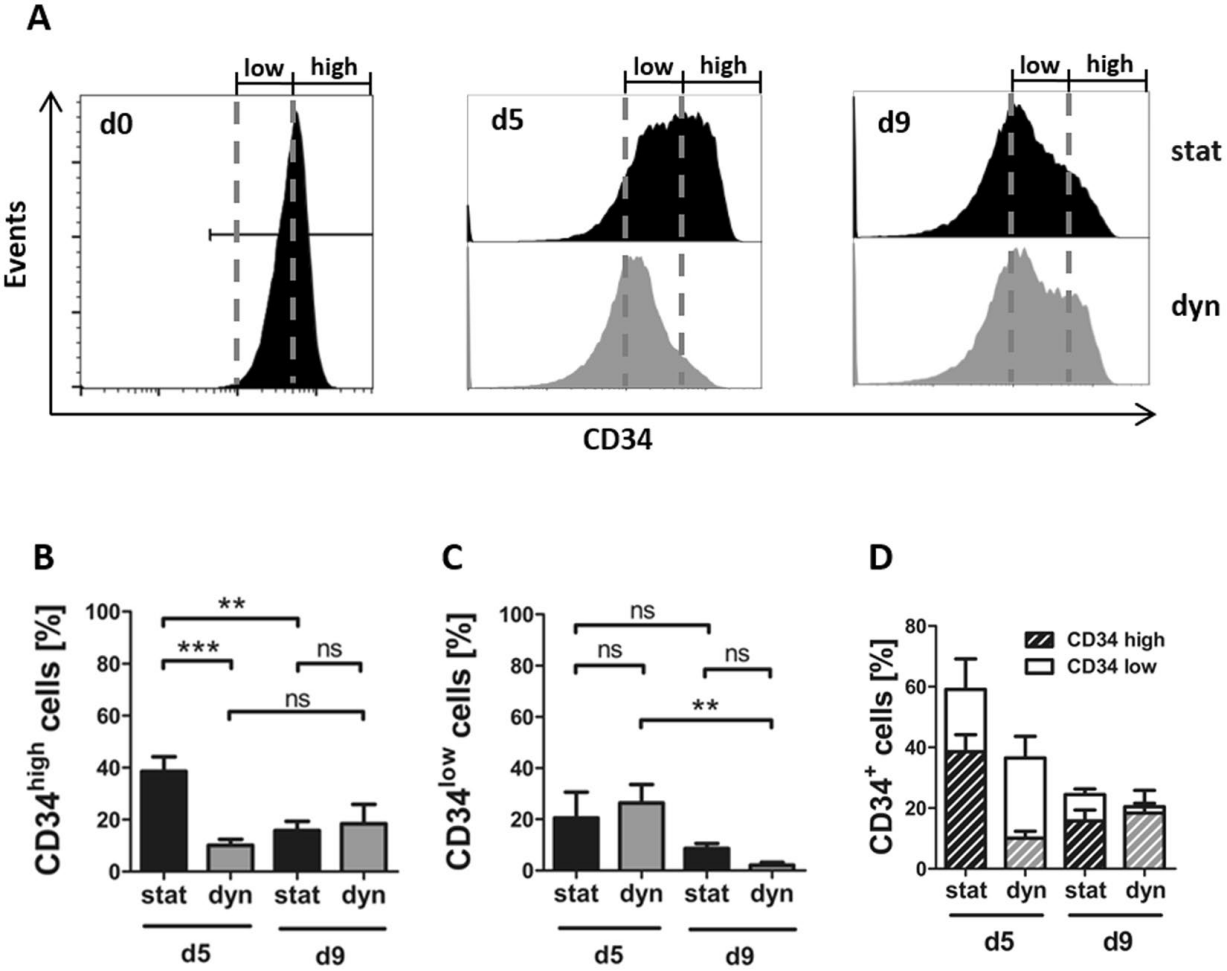
Static culture preserves a greater pool of CD34^high^ cells compared to dynamic culture in the early phase of culture. Comparison of percentages of CD34^high^ and CD34^low^ cells obtained from static and dynamic culture on day 5 and day 9. (**A**) Representative flow cytometry plots of CD34 expression analysis. Mode of culture is given on the right, the gates determining CD34^low^ and CD34^high^ cells are given on top. (**B**) Percentages of CD34^high^ and (**C**) CD34^low^ cells as assessed by flow cytometry. (**D**) Stacked column chart showing the percentage of CD34^+^ cells within the harvested cells subdivided into the percentages of CD34^low^ and CD34^high^ cells. White bars give the percentage of CD34^low^ cells, striped bars the percentage of CD34^high^ cells. (**A–D**) N = 4 independent experiments.

The effect of perfusion on CD34 expression was not only visible in the overall percentage of CD34^+^ cells but also by the appearance of subpopulations with different CD34 expression intensities (Fig. 2B). Therefore, CD34^+^ cells were subdivided into CD34^low^ and CD34^high^ cells (Fig. 3A). The static culture resulted after 5 days in a significantly larger fraction of CD34^high^ cells (38.6%) than the dynamic culture, in which only 10% of the harvested cells were CD34^high^ (Fig. 3B). Hence, the static culture contained a larger fraction of cells that resembled freshly isolated HSPCs in terms of their CD34 expression levels than the dynamic culture.

This effect was absent on day 9 when the fraction of CD34^high^ cells in the static culture was significantly reduced in comparison to day 5 and leveled to numbers similar to the ones found after dynamic culture with 15–20% of CD34^high^ cells. The CD34^low^ subpopulations were not significantly altered by perfusion of the 3D scaffolds, neither on day 5 nor on day 9 (Fig. 3C). When looking at the sum of CD34^high^ and CD34^low^ cells obtained after 5 days (Fig. 3D) it became evident that the effect of perfusion on the percentage of CD34^+^ cells (Fig. 2A,B) on day 5 was elicited within the subpopulation of CD34^high^ cells.

### Effect of perfusion on early myeloid progenitors and differentiation

**Figure 4:**
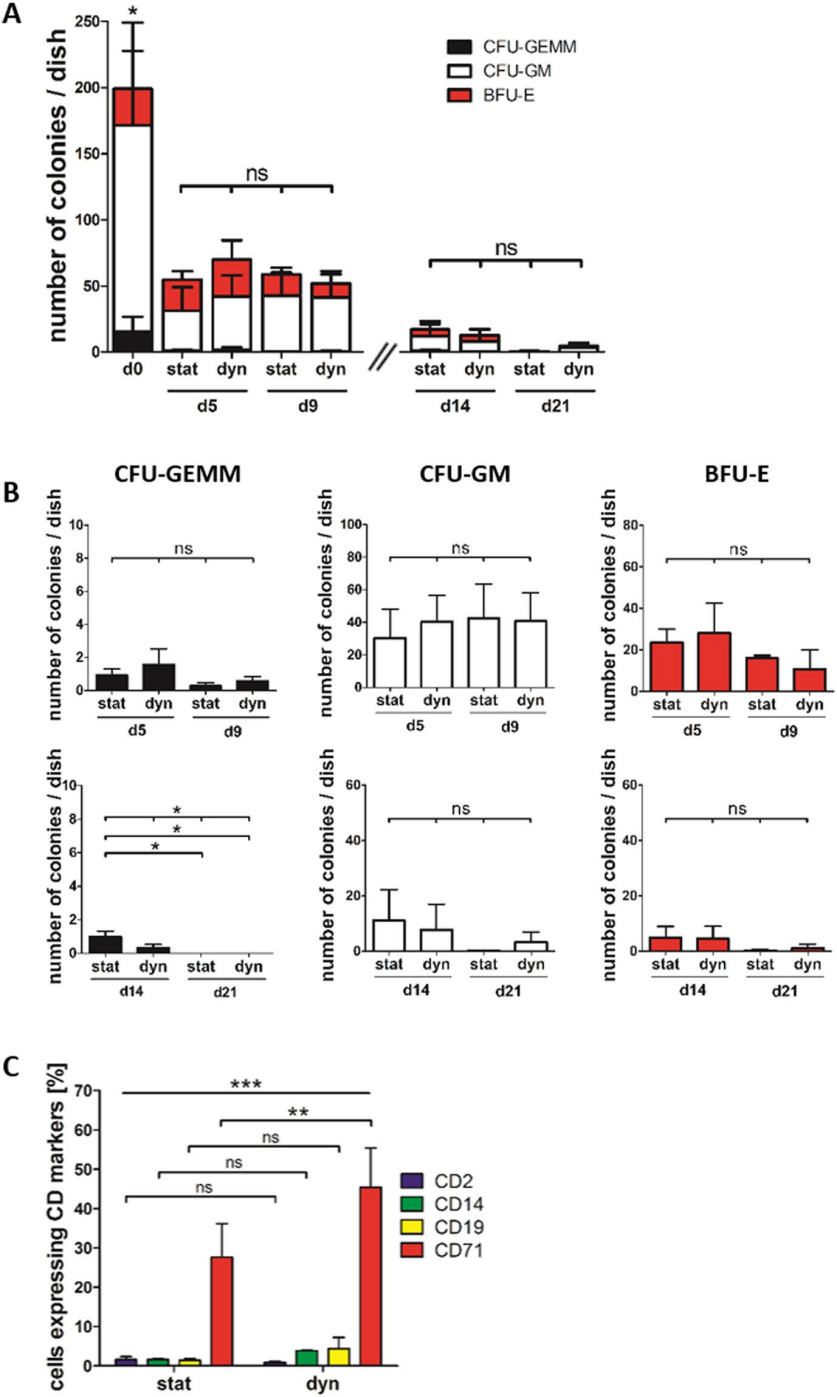
Impact of static and dynamic culture on HSPC differentiation capacity. (**A**,**B**) CFU assay of 500 cells directly after HSPC isolation from UCB (d0) or after 5, 9, 14 or 21 days of static or dynamic culture. (**A**) Summary of generated colony types and frequencies for each sample. Significance was tested based on total number of produced colonies. (**B**) Overview of generated colonies for each colony type separately (**C**) Percentages of cells expressing either CD2, CD14, CD19 or CD71 (markers for T-cells, myeloid cells, B cells and megakaryocyte/erythrocytes, respectively) as assessed by flow cytometric analysis of HSPCs after 9 days of static or dynamic culture. (**A**,**B**) Day 0, day 5 and day 9: N = 4 independent experiments; day 14 and day 21: N = 3 independent experiments; (**C**) N = 3 independent experiments, significance was tested with paired, two-tailed student’s t-test.

The number of myeloid progenitors obtained after culture was determined via colony forming unit ﻿(CFU) assays. Only early myeloid progenitors give rise to colonies in this assay, which allows retrospective determination of their number within a cell population. Similar colony frequencies were found after static and dynamic culture at all time points, indicating that comparable numbers of myeloid progenitors were present in both culture systems (Fig. 4A). Similarly, there was no difference in the number of the individual colony types detectable on day 5 and 9 (Fig. 4B, upper panel). After 14 and 21 days significantly different numbers of CFU-GEMM colonies were counted (Fig. 4B, lower panel). This difference, however, was elicited by the loss of CFU-GEMM over culture time and not by the culture condition. Therefore, it appeared that perfusion of the scaffolds had no effect on the number of early myeloid progenitors.

To study the effect of perfusion on terminal hematopoietic differentiation, cells were stained for the lineage markers CD2 = T cells, CD14 = myeloid cells, CD19 = B cells and CD71 = erythroid cells after 9 days of static or dynamic culture. Significantly more cells expressing CD71 were detected in the dynamic than in the static culture (Fig. 4C).

### The role of cytokine dose in static and dynamic culture

**Figure 5:**
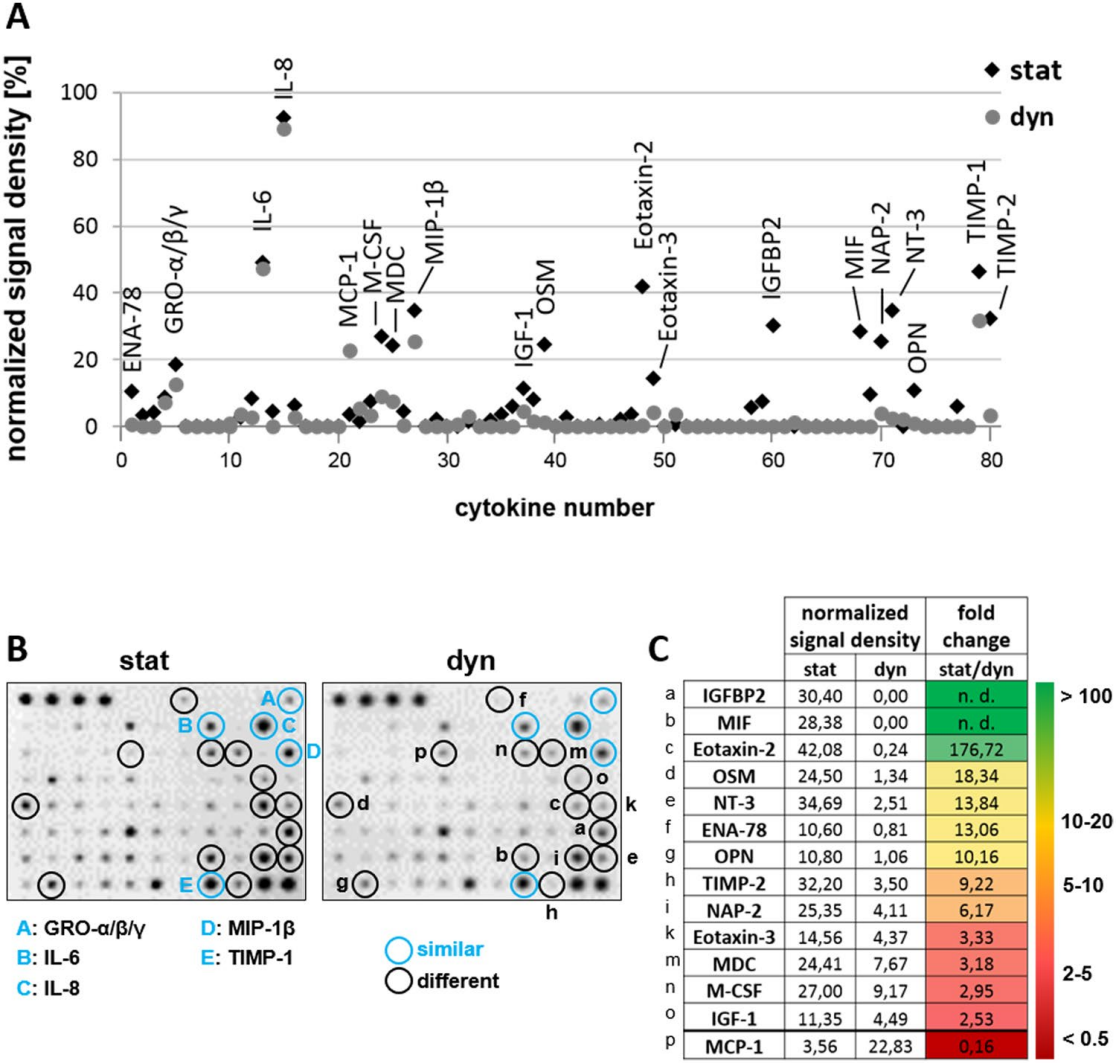
Hydrogels in static culture accumulate higher concentrations of cytokines than in dynamic conditions. The abundance of cytokines within macroporous hydrogels after 9 days of static or dynamic culture was analyzed with a human cytokine antibody array. (**A**) Normalized signal densities for individual cytokine antibody arrays probed with medium recovered from hydrogels after 9 days of static or dynamic culture. Marks with the same position on the x-axis represent signals for the same cytokine in both arrays. Marks of spots eliciting 10% signal density or more are labeled with the respective cytokine name. Black marks: cytokines within medium from static hydrogels, grey marks: cytokines within medium from dynamic hydrogels. (**B**) Optical images of the antibody arrays probed with medium from static or dynamic culture. Mode of culture is given at the top. Black circles identify cytokines with differential abundance in static and dynamic conditions. Blue circles identify cytokines that are present to similar levels in both samples. Corresponding cytokine names are given at the bottom or in the table in (C). (**C**) Summary of all cytokines that were identified to be present at different levels in static and dynamic culture inside the hydrogels. The table gives the raw values of normalized signal densities for individual cytokines in the static and dynamic culture as well as the resulting fold change stat/dyn. The letter code on the left assigns the cytokines to their corresponding location on the array shown in (**B**). The color code on the right gives a rough estimate of the abundance of the respective cytokines in the static to the dynamic culture; from dark red (fold change <1) to green (fold change >100, or not determinable (n.d.), as the cytokine is only present in the static culture mode).

To study the effect of perfusion on the availability of cytokines inside the macroporous scaffolds, medium was recovered after 9 days of culture from the hydrogels by centrifugation and screened for the presence of 80 different cytokines with an antibody array. 19 cytokines were identified to be present in the medium after static or dynamic culture or in both conditions (Fig. 5A). Five cytokines, namely GRO-α/β/γ, IL-6, IL-8, MIP-1β and TIMP-1 were found in both cultures. Amongst these five cytokines, IL-6 and IL-8 showed the highest abundance. Another 13 cytokines were predominantly present in the scaffolds after static culture and only one cytokine was found to be more abundant after dynamic culture, as indicated by the ratio of the signal density of the respective cytokines in static to dynamic culture (=fold change static/dynamic) (Fig. 5B,C). In summary, these results showed that after static culture more cytokines were found in higher abundance inside the 3D scaffolds than after dynamic culture.

### Application of the niche model under static and dynamic conditions as *in vitro* model for testing myelotoxicity

**Figure 6:**
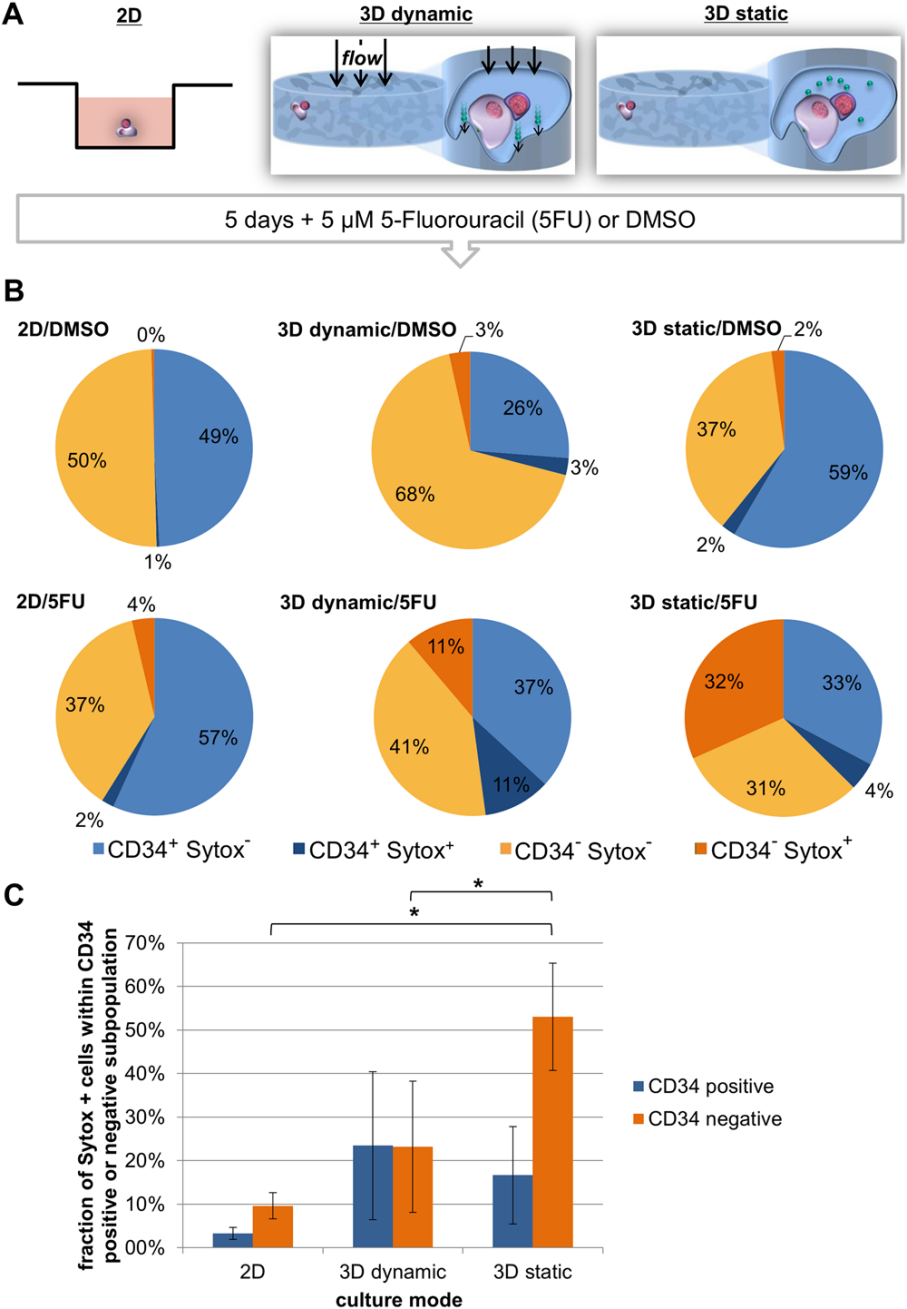
The culture mode affects the sensitivity of HSPCs to the myelotoxic drug 5-fluorouracil. (**A**) Schematic representation of the experiment. HSPCs were cultured together with MSCs in standard 2D tissue culture plates or in macroporous 3D scaffolds under static or dynamic conditions in the presence of 5-fluorouracil (5FU) or DMSO as a control for 5 days. Cells are drawn in violet, the scaffold is represented in blue, secreted molecules in green and fluid flow is indicated by black arrows. (**B**) The response of HSPCs to the 5FU treatment by cell death was assessed by Sytox staining and the results are presented as circle diagrams. Percentage of CD34^+^ cells is depicted in blue, percentage of CD34^−^ in orange, live (Sytox^−^) cells in light color and dead or late apoptotic cells (Sytox^+^) in dark blue or dark orange. (**C**) The percentage of Sytox^+^ cells within the CD34^+^ (blue) and CD34^−^ subpopulation (orange) of cells after 5 days in the presence of 5FU under the different culture conditions are presented as bar graph with error bars (mean ± standard deviation). N = 3 independent experiments.

The influence of the dimensionality (2D or 3D) and the culture mode (static or dynamic) on the susceptibility of HSPCs to chemotherapeutics was assessed by treating the cells with 5-fluorouracil as a model drug. The HSPC/MSC co-cultures were either maintained under conventional cell culture conditions in a standard 2D well plate or in the 3D scaffold under perfusion (3D dynamic) or without perfusion (3D static) in the presence or absence of 5-fluorouracil. The experimental design is schematically illustrated in Fig. 6A. After 5 days of culture the percentage of CD34^+^ cells was tested along with the percentage of dead or late apoptotic cells, which are Sytox^+^. Under control conditions, in which only the solvent DMSO was added, the percentage of dead cells was between 1% and 6%. When adding the chemotherapeutic 5-fluorouracil, the response to it was the lowest under standard 2D cell culture conditions with on average 6% of dead or late apoptotic cells. Roughly 22% of the cells died in response to 5-fluorouracil under 3D dynamic conditions and under 3D static conditions the cells appeared to be most sensitive to the chemotherapeutics treatment with 36% of dead or late apoptotic cells (Fig. 6B). Furthermore, when comparing the susceptibility of the CD34^+^ and CD34^−^ subpopulations to 5-fluorouracil under static and dynamic conditions, we found that under static conditions significantly more cells died in the CD34^−^ subpopulation, while under dynamic conditions the response of CD34^+^ and CD34^−^ cells was comparable. Therefore, it appears that the dimensionality and mode of culture affect HSPC sensitivity to 5-fluorouracil.

## Discussion

HSCs have great potential for regenerative medicine and are one decisive parameter for finding the optimal dose for chemotherapy of cancer patients. *In vitro* systems that allow mimicking the HSC niche under homeostasis and activated conditions would be a great step towards unlocking the potential of HSCs for transplantation as well as creation of *in vitro* drug testing platforms for myelosuppression during chemotherapy.

In order to study the significance of soluble niche factors in conjunction with material parameters for mimicking the niche under steady-state (stem cell-maintaining) and active (differentiating) conditions, we integrated a cell-laden, macroporous hydrogel that reflected crucial biological and physical parameters of the HSC niche^28^ into a perfusion system.

We found that during the first five days of culture the static culture yielded a larger fraction of CD34^+^ cells but similar frequencies of progenitors (CFUs) compared to the dynamic culture. The fraction of CD34^+^ cells that accounted for the observed differences could be narrowed down to the CD34^high^ cell population. Not all HSPCs that express CD34 give also rise to colonies in the CFU assay—only the early committed myeloid progenitors do so—neither lymphoid progenitors nor more innate stem and progenitor cells are detectable in this type of assay^59^. Therefore, it appears that either very early or lymphoid progenitors are maintained to a higher extent in the static and lost in the dynamic culture. Furthermore, an elevated fraction of cells harvested from the dynamic culture was found to express CD71, which shows that perfusion stimulated the onset of erythroid differentiation. Earlier studies on the effect of perfusion on HSPCs did not investigate differentiation but found that perfusion enhanced HSPC expansion^60–62^. This seems to be in contrast to the finding of the current study that perfusion enhances rather differentiation than HSPC maintenance and expansion during the first days of culture. Nevertheless, the static and dynamic culture setups, inoculated cell populations and media formulations in those earlier studies were not identical to the ones in the present study and—more importantly—no biomimetic 3D macroporous biomaterials were used for culturing HSPCs. In a recent study Berry *et al*. modelled different culture modes (no media exchange, media exchange, fed batch and perfusion) for HSPC cultures and predicted that media exchange is the most effective strategy to expand HSPCs^63^. As we performed regular medium exchange in the static culture setups, this prediction fits well to our findings.

The observed differences in HSPC maintenance and differentiation under static and dynamic conditions could be elicited by physical and biochemical parameters that are changed by the fluid flow. These are predominantly shear stress and concentration profiles of soluble stimulatory or inhibitory molecules, respectively. To evaluate the potential contribution of shear stress to the observed effects, computational fluid dynamics were carried out that revealed occurring shear stresses from ~3.5 to a maximum of 7 × 10^−4^ Pa. These values are ≥3 orders of magnitude smaller than the threshold described for mechanostimulatory effects of shear stress on cells residing in the bone marrow, including mesenchymal stromal cells (0.9 Pa), osteoblasts (2.0 Pa) as well as cells deriving from the hematopoietic lineage such as osteoclasts (0.5 to 2.0 Pa) or mature (1.0 to 2.0 Pa) and immature megakaryocytes (0.15 to 0.4 Pa)^64–68^. Shear stress was reported to have an effect on the development of HSCs during embryonic development. However, also in this context the estimated effective wall shear stresses were around 0.5 Pa^69^. *In vitro*, effects of fluid dynamics on the expansion of mononuclear cells from UCB (containing HSPCs) in spinner flasks could be observed at shear stresses of ~0.1 Pa^70^. Therefore, it appeared that the shear stress occurring in the present study in the artificial bone marrow analog during perfusion was too small to effectively stimulate cells mechanically. Although combinatorial effects of shear stress and the other parameters could not be excluded and our assumptions might thus not be rigorous, this analysis led to the conclusion that the observed effects of perfusion on HSPCs were elicited by the change of concentration profiles of soluble biochemical factors that arose with fluid flow.

Under this assumption our results suggested that during the first five days of culture the regular medium exchange in the static culture mode was sufficient to wash out secreted inhibitory signals from the scaffold and to maintain at the same time a sufficiently high concentration of stimulatory signals. Under perfusion both inhibitory and, probably more important, stimulatory signals may have been washed out of the scaffolds. To address this hypothesis, we analyzed the cytokine profiles within the hydrogels. This analysis revealed lower cytokine abundance within the hydrogels with than without perfusion. This finding is not surprising since the flow of medium caused by perfusion rapidly dilutes the released cytokines in the entire volume (10 ml) of medium in the perfusion reservoir. In the static culture, medium exchange within the 3D scaffold is mainly dependent on diffusion. Thereby, medium and cytokine exchange is much slower in the static than in the dynamic culture with perfusion. This allowed a higher accumulation of cytokines within the macroporous hydrogels in the static mode. Together with the finding that after 5 days of static culture a higher fraction of  CD34^high^ cells could be detected, this result suggests that a higher availability of cytokines in the scaffold without perfusion supported the maintenance of CD34^+^ cells. A number of cytokines that were found to be differential in their abundance in the scaffolds after static or dynamic culture were reported to have an impact on the behavior of HSPCs or their hematopoietic progenies (Table 1). The two cytokines that showed the strongest enrichment in the static medium—IGFBP2 and MIF—are known to be beneficial for the specific, targeted expansion of HSPCs or general HSPC proliferation *ex vivo* (Table 1), respectively^71–76^. This underscores the conclusion that the stimulation by accumulated cytokines might be the reason for the supportive effect of the static culture mode on CD34^+^ cell maintenance.

**Table 1:** Described effects of differentially abundant cytokines on HSPCs or hematopoietic cells.

cytokine	described effects on HSPCs or differentiated hematopoietic cells
IL-6	crucial for survival and self-renewal of HSPCs^97^ ^a^
IL-8	induces mobilization of HSPCs^98^ ^a^
GRO-β	mobilization of early HSPCs^99^ ^a^
IL-6 + IL-8 + GRO-α/β/γ	combination stimulates *ex vivo* expansion of HSPCs^100^ ^b^
IL-8, GRO-α, MCP-1	individually mediate adhesion of differentiated hematopoietic cells^101^ ^b^
TIMP-2	has erythroid-potentiating activity^102^ ^b^
IGFBP2 (+IGFBP1 + Ang-1)	supports expansion of HSPCs *ex vivo* ^71, 72^ ^b^; ^73, 74^ ^a^
MIF	induces proliferation of fully differentiated hematopoietic cells^103^ ^b^; is a lymphoma growth factor^75^ ^a^
OPN	limits proliferation of HSPCs^104^ ^a^

^a^In the murine system. ^b^In human system.

Amongst the 14 differentially abundant cytokines a remarkable number of chemoattractants was identified: GRO-α/β/γ (CXCL1/2/3), IL-8 (CXCL8), MIP-1β (CCL4), Eotaxin-2 (CCL24), Eotaxin-3 (CCL26), ENA-78 (CXCL5), NAP-2 (CXCL7) and MCP-1 (CCL2). As the name “chemoattractant” implies, these factors direct cell movement towards sites of local concentration. Those chemoattractive cytokines detected inside the hydrogel might favor the persistence of HSPCs inside the hydrogel by impeding the migration of HSPCs out of the hydrogel. MIF and OPN are two cytokines that were reported to be upregulated in hypoxic conditions^77, 78^. Since both were detected in the static culture medium but not or to negligible amounts in the dynamic one, this leads to the conclusion that the HSPCs and MSCs within the scaffolds consume oxygen faster than the passive oxygen transport could compensate in the static culture mode, resulting in hypoxic areas inside the hydrogel. In the dynamic culture, the continuous flow of fresh medium through the hydrogel prevents hypoxia. Therefore, it appears that a combination of a lower oxygen but higher cytokine availability influenced HSPC behavior in the static culture towards an enhanced maintenance of CD34^+^ cells. This conclusion fits to previous reports showing that hypoxia supports HSC maintenance and quiescence^35, 79–81^ and that the availability of soluble factors that enables autocrine and/or paracrine signaling is an important determinant in HSC cultures^82^, particularly in biomaterial-approaches in which the cells are maintained in confined spaces^83^. Perfusion, in contrast, seemed to foster hematopoietic differentiation within the 3D scaffolds, as seen by the expression of CD71, a marker specific for erythroid progenitors. Possible explanations for the enhanced erythroid differentiation under perfusion might be the lack of maintenance-supporting cytokines or the high oxygen concentration (~21%) within the scaffold in this culture mode. Oxygen concentration was shown previously to play a role in *in vitro* erythropoiesis^84^. All in all, it appears that during the first days of culture perfusion optimized the conditions for hematopoietic differentiation—mimicking the niche in an activated state—while the static culture mode fostered the maintenance of CD34^high^ HSPCs—resembling the niche under steady-state homeostasis—in the simplified 3D bone marrow analog. Thereby, controlling the culture mode allows to mimic the niche in 3D models under different physiological conditions, which might be important for applications as test systems for drugs.

Interestingly, the differences observed during the first 5 and 9 days of culture, vanished at later time points. The number of harvested cells did not increase any further after day 9 and at the same time the percentage of CD34^+^ cells did not drop any further. This means that after 9 days of culture, a constant state of culture was reached, in which the cells neither proliferated nor differentiated to a larger extend. One might speculate that this upper limit of cell growth was set by the limited growth area accessible for the cells inside of the scaffold. At these later time points, the static culture setup was not superior to perfusion in terms of HSPC expansion, anymore. Because medium exchange—as carried out in the static setup—is labor intensive and hard to scale up, perfusion—as an automatable and tunable method—might be the more effective strategy to expand HSPCs at larger scale during longer periods of time. The maximum size of 3D constructs containing living cells is limited by the distance over which diffusion allows efficient nutrition/supply of the cells. Without perfusion the relative concentration of molecules within the biomaterial drops quickly with increasing distance from the biomaterial–media interface^85^. Perfusion provides the possibility to overcome this limitation of insufficient medium supply in the central parts of larger biomaterials and allows, therefore, upscaling the scaffold size. Thereby, the growth area/volume and the number of cells that can be expanded within the scaffold can be greatly enhanced.

In order to evaluate the potential of the presented system as *in vitro* test system for assessing myelotoxicity, the chemotherapeutic 5-fluorouracil was applied, which is known to affect the hematopoietic system^56, 86^. The concentration of 5 µM was used as this concentration is in the range of IC_50_ value of 5-fluorouracil found in CFU-GM assays, which proved to be predictive for human myelosuppression^87–89^. As seen before in other studies, also the present study showed that cells in 3D are more sensitive to toxic effects of chemicals or chemotherapeutics than in 2D^87, 90, 91^. The presented results show that the toxicity of 5-fluorouracil was strongly underestimated when tested in 2D. In the 3D cultures more cells died in response to 5 µM 5-fluorouracil than in 2D. This finding fits well with reports from other studies that observed that HSCs or leukemic cells respond differently to anti-cancer treatments in 3D than in 2D. One study showed that human acute myeloid leukemia cells exhibit stronger resistance to the tested drugs when cultured in a 3D setup as compared to cells from a 2D suspension culture^38^. It was also reported that murine HSCs cultured in a 3D bone marrow-on-a-chip responded to γ-radiation in a comparable manner as HSCs *in vivo* ^55^. In the present study the toxic effect was most pronounced under 3D static conditions with 36 ± 10% (mean ± standard deviation) of hematopoietic cells that were affected. These values approached the half-maximal efficacy (50%) that was observed in previous studies for testing myelotoxicity with the help of CFU-GM assays at this concentration^87, 88, 92^. While CFU assays focus on the myeloid lineage of hematopoietic cells and do not allow to reflect different physiological conditions, the system described here allows investigating the entire population of CD34^+^ cells and their descendants as well as mimicking a more static and activated state. Interestingly, analyzing the effects of 5-fluorouracil on the CD34^+^ subpopulation of cells—which are assumed to be more native—and the more mature CD34^−^ subpopulation, revealed that the static and the dynamic conditions strongly affected the susceptibility of the different subpopulations to the toxicity of 5-fluorouracil. While in the static culture mode CD34^−^ cells were more sensitive to 5-fluorouracil treatment, under dynamic condition both subpopulations responded similarly. The mechanism of action of 5-fluorouracil implies that primarily metabolically active and cycling cells are attacked^86^. In the HSC niche under homeostatic conditions, the progeny rather than the dormant stem cells are proliferative and metabolizing, which might explain the results observed in the static bone marrow analog. During hematopoietic stress situations as they occur during chemotherapy the stem cell compartment wakes up and gets activated^93, 94^. Thereby, the prevalent activity—and thus susceptibility to 5-fluorouracil—of more mature cells might be abrogated. These results indicate that the presented system is applicable as drug testing system as it is able to reproduce the results available with the established and approved CFU-GM test. However, in addition to this test the system is not limited to the myeloid lineage and allows investigating effects of drugs not only during steady state but also in alarm situations.

## Conclusion

Our current study showed that by integrating a biomimetic 3D bone marrow analog into a perfusion system, different processes that occur *in vivo* in the bone marrow can be mimicked: maintenance and differentiation of CD34^+^ cells. Thereby, this setup allows directing the outcome of HSPC *in vitro* culture at early time points of culture via the culture mode, reflecting HSPC behavior in the niche under different physiological conditions. At later time points perfusion was as effective as the static culture setup in maintaining CD34^+^ cells, which is important for future biomaterial-based approaches to expand HSPCs for clinical applications, as it allows upscaling and automatization. Finally, we performed proof-of-concept experiments that demonstrate that the presented system opens new avenues to use the developed 3D model of the bone marrow niche for drug screening and toxicity testing, in particular for myelotoxic effects of chemotherapeutics. All in all, the presented method of a perfused 3D model of the HSC bone marrow niche appears to be a versatile tool (i) to improve HSC culture for clinical applications of the stem cells and their progenies and (ii) to develop human 3D *in vitro* bone marrow analogs for pharmaceutical toxicity testing.

## Materials and Methods

### Cell culture

Human CD34^+^ HSPCs were isolated from UCB. The blood was obtained from the DKMS Cord Blood Bank (Dresden, Germany) or the Mannheim Cord Blood Bank (Mannheim, Germany) with informed consent of the parents and approval by the local ethics committee (Ethik-Kommission bei der Landesärztekammer Baden-Württemberg; B-F-2013-111). Mononuclear cells were isolated from UCB by density gradient centrifugation. CD34^+^ cells were isolated with the help of magnetic activated cell sorting (MACS, Miltenyi Biotec GmbH, Bergisch Gladbach, Germany) according to the manufacturer’s instructions. Purity of the freshly isolated cells was tested via flow cytometry—as described below—and the cells were only used if a minimum of 95% of the cells were positively stained for CD34^+^. HSPCs were maintained in serum-free HPC Expansion Medium DXF (PromoCell GmbH, Heidelberg, Germany) supplemented with Cytokine-Mix E (containing TPO, SCF, Flt-3 ligand and IL-3) and 1% (v/v) penicillin/streptomycin (Sigma-Aldrich, Taufkirchen, Germany).

Mesenchymal stem/stromal cells (MSCs) were isolated, expanded and characterized as described before^95^. Briefly, mononuclear cells were isolated using a Ficoll-gradient, then plated in DMEM low glucose (Sigma-Aldrich) supplemented with 10% (v/v) FBS (Sigma-Aldrich) and 1% (v/v) penicillin/streptomycin. At subconfluent stages, MSC were passaged and seeded at a densitiy of 200 cells/cm^2^. Cells were characterized according to ISCT criteria: fibroblastoid morphology, specific immune phenotype, adipogenic and osteogenic differentiation potential, as well as suppression of T cell proliferation^95^. Cells up to passage 6 were subjected to experiments.

### Hydrogel fabrication

Macroporous hydrogels were made from PEG-diacrylate as scaffolding material and NaCl as porogen via a salt leaching technique as described previously^28, 50^. Briefly, a 33% (w/v) PEG-diacrylate (M_n_ = 6,000) solution was prepared by dissolving 333 mg PEG-diacrylate in 1 mL saturated aqueous NaCl solution. As bioactive compound that allows cell adhesion to the final hydrogel, 40 µL of a 500 µM RGDSK-PEG_6_-acrylate (referred to here as RGD, kindly provided by Hubert Kalbacher, University of Tübingen, Germany) were added to the solution. 600 mg size selected NaCl crystals (Merck, Darmstadt, Germany) with sizes between 40 and 100 µm were added to 150 mg of the PEG-diacrylate/RGD solution (final porogen concentration: 80% (w/w)). 750 mg of this solution were transferred to a well of a 48 well plate (Greiner Bio-One, Frickenhausen, Germany) and 45 µL ammonium persulfate (APS; AppliChem, Darmstadt, Germany) as well as 8 µL Tetramethylethylenediamine (Temed; Merck, Darmstadt, Germany) were added which initiated chemical polymerization and crosslinking of the PEG-diacrylate and the acrylated RGD peptide. The resulting gel was incubated in 800 mL of water for 3 days for swelling the hydrogel and leaching out the NaCl crystals resulting in a macroporous RGD-PEG-diacrylate hydrogel. Prior to experiments, the hydrogels were dehydrated by incubation in an ethanol series with increasing concentrations of ethanol (50% (v/v), 60% (v/v), 70% (v/v), 80% (v/v), 90% (v/v), 2 × 100% (v/v)) in water for 10 min each. Dehydrated hydrogels were frozen in 100% (v/v) ethanol at −80 °C for 2 days and then freeze dried (Christ Gefriertrocknungsanlagen, Osterode am Harz, Germany) for 24 hours.

### Colonization of the macroporous hydrogels with cells

Directly prior to cell experiments, freeze-dried hydrogels were sterilized by UV-irradiation for 10 min from both sides. For each hydrogel, 500,000 MSCs and 125,000 HSPCs were combined and washed with PBS. Cells were pelleted and resuspended in 50 µL HPC medium. For cell seeding, 25 µL of the cell suspension were pipetted dropwise onto the hydrogel followed by 10 min incubation, during which the hydrogel swelled and soaked up the cell suspension. The hydrogel was turned around and seeded from the opposite site by applying the remaining 25 µL of the cell suspension. Cells were allowed to adhere for 20 min before the hydrogel was transferred to a well of a 24 well plate with 2 mL of HPC medium. Seeded hydrogels were incubated at 37 °C and 5% CO_2_ over night in order to ensure efficient adhesion of the cells to the scaffolds.

### Static and dynamic culture inside the 3D macroporous hydrogel

For the static culture condition, half of the medium was exchanged 5 times a week by fresh HPC medium and the well plate was incubated without agitation at 37 °C and 5% CO_2_.

To achieve dynamic culture conditions, in which the scaffolds were perfused with medium, the colonized macroporous hydrogels were combined with a bioreactor. For that purpose, the colonized scaffolds were punched to discs of 11 mm in diameter and transferred to a perfusion reactor 16 hours after colonization. The perfusion reactor was developed by the group of Jan Hansmann^96^. The reactors were connected to previously sterilized PharMed^®^ BPT SC0740 tubing with 0.25 mm inner diameter (Ismatec, Wertheim, Germany) and a customized 20 ml Schott bottle as medium reservoir containing 10 ml of fresh HPC medium. The Schott bottle was ventilated through a 0.2 µm sterile filter. For experiments proceeding over 21 days, 3 ml of the expended medium were exchanged with 5 ml of fresh HPC medium on day 7 and another 5 ml were exchanged on day 14. The medium was pumped through the tubing, reactor and scaffold with a MCP CA4 peristaltic pump (Ismatec, Wertheim, Germany) with flow rates of 60 µL/min. Pump and reactor were kept inside an incubator at 37 °C and 5% CO_2_ in a humidified atmosphere. The medium was pumped from the medium reservoir via the tubing through the hydrogel scaffold back to the medium reservoir, from where it was constantly recycled.

After 5, 9, 14 or 21 days of culture cells were harvested from the macroporous scaffolds as described before^50^. Briefly, the scaffolds were cut in pieces, trypsinized, shaken and cells were collected via centrifugation. Harvested cells were counted. Cell numbers were corrected for the determined harvesting efficiency (91%) and the ones from bioreactor setups were also corrected for the initial cell loss that occurred by punching out the hydrogels. The cell numbers obtained at the different time points differed not significantly between perfused and non-perfused hydrogels (Fig. 2C). Thus, cell loss due to perfusion was neglected. The harvested cells were analyzed for CD34 expression and viability via flow cytometry as well as their colony forming potential. Furthermore, cells retrieved at culture day 9 were analyzed for the expression of differentiation markers via flow cytometry.

### 5-fluorouracil treatment

To assess the impact of the culture mode on the cells’ sensitivity to chemotherapeutics, HSPCs were cultured together with MSCs for 5 days under standard cell culture conditions (2D) or within the macroporous 3D scaffolds as described above under static and dynamic conditions in the presence or absence of 5 µM 5-fluorouracil (Merck, Darmstadt, Germany). A stock solution of 5 mM 5-fluorocuracil in DMSO was prepared and added in 1:1000 dilution to the cell culture medium. The solvent DMSO was used as a negative control. No medium exchange was carried out. After 5 days cells were harvested and cell death as well as CD34 expression were assessed by flow cytometry as described below.

### Back Pressure measurements

A pressure sensor (HJK, Merching, Germany) was introduced into the setting of the dynamic culture behind the pump and before the perfusion reactor. The occurring back pressure was monitored for increasing pump speed in 5 rpm steps.

### Computational fluid dynamics

To investigate hydrogel permeability and shear stress distribution in the scaffold, computational fluid modeling was performed using COMSOL Multiphysics (COMSOL Multiphysics GmbH, Göttingen, Germany). Briefly, five three-dimensional domains comprising an inlet, an outlet, a porous media domain representing the hydrogel, and two domains for perforated metal plates were generated according to the bioreactor geometry using SolidWorks (Dassault Systemes, Stuttgart, Germany). To induce fluid flow, laminar inlet and ambient pressure outlet conditions were applied to the respective fluid domains and Darcy’s law derived for creeping flows was applied to the porous media domain. The hydrogel porosity was set to 80% according to previous work^28^. Hydrogel permeability was obtained by parametric sweep while considering pressure measurements at increasing flow rates. Shear stress data was collected by probing the hydrogel domain.

### Flow Cytometry

The expression of CD34 and the lineage markers CD14, CD19 and CD71 of the harvested cells was assessed via flow cytometry. 20,000–100,000 cells were stained with anti-CD34-FITC (clone 581, Life Technologies), anti-CD2-FITC (clone HIT11, Immunotools, Friesoythe, Germany), anti-CD14-PerCP (clone 18D11, Immunotools), anti-CD19-PE/Dye647 (clone LT19, Immunotools) or anti-CD71-PE/Dy647 (clone MEM-75, Immunotools) and the respective isotype controls. CD34-labelled cells were additionally stained with the dead cell stain SytoxAADvanced (LifeTechnologies) according to the manufacturer’s instructions to determine viability and to exclude dead cells from the analysis.

Staining was performed for 45 min at 4 °C. Measurements were carried out with an Attune^®^ Accoustic Flow Cytometer (Applied Biosystems, Darmstadt, Germany). Data were analyzed using FlowJo (TreeStar, Ashland, OR, USA).

### Colony forming unit assay

Colony forming unit (CFU) assays were prepared on day 0, 5, 14 and 21 of culture in order to retrospectively asses the myeloid differentiation potential of the harvested cells. 1500 cells were resuspended in 300 µl IMDM + 2% FBS (Stemcell Technologies SARL, Grenoble, France) and then mixed with 3 ml Methocult H4434 Classic (Stemcell Technologies). 1.1 ml of this cell suspension were plated in triplicate into 35 mm Petri dishes (Greiner Bio-One, Frickenhausen, Germany) and incubated in a wet chamber at standard cell culture conditions. After 12 days the colonies were counted and individual colonies were assigned to one of the following colony types: CFU-GEMM (granulocyte, erythroid, monocyte & megakaryocyte progenitors), CFU-GM (granulocyte & macrophage progenitors) or BFU-E (erythroid progenitors).

### Cytokine Antibody Array

A cytokine antibody array was conducted to compare the abundance of cytokines inside of macroporous hydrogels after static and dynamic culture. For this purpose, the medium from the interior of the macroporous hydrogels was collected by centrifugation after 9 days of culture. The samples collected from 4 independent experiments were combined and analyzed for the presence of secreted cytokines with the human cytokine antibody array C5 (RayBiotech, NorCross, GA, USA) detecting 80 different cytokines following the manufacturer’s manual. Each array was incubated with 70 µl of medium containing 210 µg protein. Freshly prepared medium was analyzed aside the samples as background control. The arrays were evaluated with densiometry using ImageJ software (http://rsbweb.nih.gov/ij/). Data analysis was done as follows: Negative control spots (no antibodies on membrane) served as background and their signal was subtracted from the signal of remaining spots. For normalization within individual arrays, signal of negative control spots was set to 0% and signal of positive control spots was set to 100% intensity. In order to remove medium inherent signal, the signal of spots from the medium control was subtracted from the signal of corresponding spots of the arrays probed with medium from the static or dynamic culture. The fold change for static/dynamic was calculated for cytokines that exhibited at least in one condition a signal intensity of 10% or higher. Differences of cytokine abundance in static and dynamic conditions were assumed if fold changes were higher than 2 or smaller than 0.5.

### Statistical Analysis

Data are presented as mean ± standard deviation, unless stated otherwise. Differences between groups were tested with one-way ANOVA and Tukey-Kramer post-hoc test. Differences between two samples were tested with paired, two-tailed Student’s t-tests. Differences were considered significant if p ≤ 0.05. Significances are given as follows: * for p ≤ 0.05; ** for ≤ 0.01; *** for p ≤ 0.001 and ns (not significant) for p ≥ 0.05.

## Electronic supplementary material

Supplementary Figures

## References

[1] Bryder D, Rossi DJ, Weissman IL (2006). Hematopoietic stem cells: the paradigmatic tissue-specific stem cell. Am J Pathol.

[2] Eaves CJ (2015). Hematopoietic stem cells: concepts, definitions, and the new reality. Blood.

[3] Rieger MA, Schroeder T (2012). Hematopoiesis. Cold Spring Harb Perspect Biol.

[4] Porada CD, Atala AJ, Almeida-Porada G (2015). The hematopoietic system in the context of regenerative medicine. Methods.

[5] Jaing TH (2014). Umbilical cord blood: a trustworthy source of multipotent stem cells for regenerative medicine. Cell Transplant.

[6] Jaing TH (2014). Successful hematopoietic reconstitution by unrelated donor cord blood transplantation in children with Fanconi anemia: report of 3 cases. J Pediatr Hematol Oncol.

[7] Scaradavou A (2013). Double unit grafts successfully extend the application of umbilical cord blood transplantation in adults with acute leukemia. Blood.

[8] Horwitz, M. E. *Ex Vivo* Expansion or Manipulation of Stem Cells to Improve Outcome of Umbilical Cord Blood Transplantation. *Curr Hematol Malig Rep* (2015). 10.1007/s11899-015-0297-7 26677145

[9] de Lima M (2012). Cord-blood engraftment with *ex vivo* mesenchymal-cell coculture. New Engl J Med.

[10] Delaney C (2010). Notch-mediated expansion of human cord blood progenitor cells capable of rapid myeloid reconstitution. Nat. Med.

[11] Peled T (2004). Linear polyamine copper chelator tetraethylenepentamine augments long-term *ex vivo* expansion of cord blood-derived CD34+ cells and increases their engraftment potential in NOD/SCID mice. Exp. Hematol..

[12] Peled T (2004). Pre-clinical development of cord blood-derived progenitor cell graft expanded *ex vivo* with cytokines and the polyamine copper chelator tetraethylenepentamine. Cytotherapy.

[13] Horwitz ME (2014). Umbilical cord blood expansion with nicotinamide provides long-term multilineage engraftment. J. Clin. Invest..

[14] Peled T (2012). Nicotinamide, a SIRT1 inhibitor, inhibits differentiation and facilitates expansion of hematopoietic progenitor cells with enhanced bone marrow homing and engraftment. Exp. Hematol..

[15] Boitano AE (2010). Aryl hydrocarbon receptor antagonists promote the expansion of human hematopoietic stem cells. Science.

[16] Wagner JE (2016). Phase I/II Trial of StemRegenin-1 Expanded Umbilical Cord Blood Hematopoietic Stem Cells Supports Testing as a Stand-Alone Graft. Cell Stem Cell.

[17] Walasek MA, van Os R, de Haan G (2012). Hematopoietic stem cell expansion: challenges and opportunities. Ann N Y Acad Sci.

[18] Morrison SJ, Scadden DT (2014). The bone marrow niche for haematopoietic stem cells. Nature.

[19] Schofield R (1978). The relationship between the spleen colony-forming cell and the haemopoietic stem cell. Blood Cells.

[20] Zhang J (2003). Identification of the haematopoietic stem cell niche and control of the niche size. Nature.

[21] Mendez-Ferrer S (2010). Mesenchymal and haematopoietic stem cells form a unique bone marrow niche. Nature.

[22] Calvi LM (2003). Osteoblastic cells regulate the haematopoietic stem cell niche. Nature.

[23] Gattazzo F, Urciuolo A, Bonaldo P (2014). Extracellular matrix: A dynamic microenvironment for stem cell niche. Biochim. Biophys.Acta: Gen. Subjects.

[24] Klein G (1995). The extracellular matrix of the hematopoietic microenvironment. Experientia.

[25] Lee-Thedieck C, Spatz JP (2012). Artificial niches: biomimetic materials for hematopoietic stem cell culture. Macromol Rapid Commun.

[26] Lee-Thedieck, C. & Spatz, J. P. Biophysical regulation of hematopoietic stem cells. *Biomaterials Science* (2014). 10.1039/c4bm00128a 32481942

[27] Leisten I (2012). 3D co-culture of hematopoietic stem and progenitor cells and mesenchymal stem cells in collagen scaffolds as a model of the hematopoietic niche. Biomaterials.

[28] Raic A, Rödling L, Kalbacher H, Lee-Thedieck C (2014). Biomimetic macroporous PEG hydrogels as 3D scaffolds for the multiplication of human hematopoietic stem and progenitor cells. Biomaterials.

[29] Ventura Ferreira MS (2012). Cord blood-hematopoietic stem cell expansion in 3D fibrin scaffolds with stromal support. Biomaterials.

[30] Jiang J, Papoutsakis ET (2013). Stem-cell niche based comparative analysis of chemical and nano-mechanical material properties impacting *ex vivo* expansion and differentiation of hematopoietic and mesenchymal stem cells. Adv Healthc Mater.

[31] Robb L (2007). Cytokine receptors and hematopoietic differentiation. Oncogene.

[32] Zhang CC, Lodish HF (2008). Cytokines regulating hematopoietic stem cell function. Curr Opin Hematol.

[33] Eliasson P, Jonsson JI (2010). The hematopoietic stem cell niche: low in oxygen but a nice place to be. J Cell Physiol.

[34] Spencer JA (2014). Direct measurement of local oxygen concentration in the bone marrow of live animals. Nature.

[35] Jez M, Rozman P, Ivanovic Z, Bas T (2015). Concise review: the role of oxygen in hematopoietic stem cell physiology. J Cell Physiol.

[36] Gurkan UA, Akkus O (2008). The mechanical environment of bone marrow: a review. Ann Biomed Eng.

[37] Fujita A (2010). Hematopoiesis in regenerated bone marrow within hydroxyapatite scaffold. Pediatr. Res..

[38] Bray, L. J. *et al*. A three-dimensional *ex vivo* tri-culture model mimics cell-cell interactions between acute myeloid leukemia and the vascular niche. *Haematologica*, [Epub ahead of print] (2017). 10.3324/haematol.2016.157883 PMC5566030 28360147

[39] Ventura Ferreira MS (2016). An engineered multicomponent bone marrow niche for the recapitulation of hematopoiesis at ectopic transplantation sites. J Hematol Oncol.

[40] Di Buduo CA (2015). Programmable 3D silk bone marrow niche for platelet generation *ex vivo* and modeling of megakaryopoiesis pathologies. Blood.

[41] Kurth I, Franke K, Pompe T, Bornhauser M, Werner C (2009). Hematopoietic stem and progenitor cells in adhesive microcavities. Integrative biology: quantitative biosciences from nano to macro.

[42] Kurth I, Franke K, Pompe T, Bornhauser M, Werner C (2011). Extracellular matrix functionalized microcavities to control hematopoietic stem and progenitor cell fate. Macromol. Biosci..

[43] Gobaa S (2011). Artificial niche microarrays for probing single stem cell fate in high throughput. Nat. Methods.

[44] Lutolf MP, Doyonnas R, Havenstrite K, Koleckar K, Blau HM (2009). Perturbation of single hematopoietic stem cell fates in artificial niches. Integr Biol (Camb).

[45] Lutolf MP, Gilbert PM, Blau HM (2009). Designing materials to direct stem-cell fate. Nature.

[46] Demange, E. *et al*. Survival of cord blood haematopoietic stem cells in a hyaluronan hydrogel for *ex vivo* biomimicry. *J Tissue Eng Regen Med* (2012). 10.1002/term.1482 22473677

[47] Lee J, Kotov NA (2009). Notch ligand presenting acellular 3D microenvironments for *ex vivo* human hematopoietic stem-cell culture made by layer-by-layer assembly. Small.

[48] Mortera-Blanco T, Mantalaris A, Bismarck A, Aqel N, Panoskaltsis N (2011). Long-term cytokine-free expansion of cord blood mononuclear cells in three-dimensional scaffolds. Biomaterials.

[49] Sharma MB, Limaye LS, Kale VP (2012). Mimicking the functional hematopoietic stem cell niche *in vitro*: recapitulation of marrow physiology by hydrogel-based three-dimensional cultures of mesenchymal stromal cells. Haematologica.

[50] Rödling L, Raic A, Lee-Thedieck C (2014). Fabrication of biofunctionalized, cell-laden macroporous 3D PEG hydrogels as bone marrow analogs for the cultivation of human hematopoietic stem and progenitor cells. Methods Mol. Biol..

[51] Choi YS, Noh SE, Lim SM, Kim DI (2010). Optimization of *ex vivo* hematopoietic stem cell expansion in intermittent dynamic cultures. Biotechnol Lett.

[52] Liu Y, Liu T, Fan X, Ma X, Cui Z (2006). *Ex vivo* expansion of hematopoietic stem cells derived from umbilical cord blood in rotating wall vessel. J. Biotechnol..

[53] Xue C, Kwek KY, Chan JK, Chen Q, Lim M (2014). The hollow fiber bioreactor as a stroma-supported, serum-free *ex vivo* expansion platform for human umbilical cord blood cells. Biotechnol J.

[54] Schmelzer E, Finoli A, Nettleship I, Gerlach JC (2015). Long-term three-dimensional perfusion culture of human adult bone marrow mononuclear cells in bioreactors. Biotechnol. Bioeng..

[55] Torisawa YS (2014). Bone marrow-on-a-chip replicates hematopoietic niche physiology *in vitro*. Nat Methods.

[56] Friberg LE, Karlsson MO (2003). Mechanistic models for myelosuppression. Invest. New Drugs.

[57] Corrie PG (2011). Cytotoxic chemotherapy: clinical aspects. Medicine (Baltimore).

[58] Kurtin S (2012). Myeloid toxicity of cancer treatment. J Adv Pract Oncol.

[59] Coulombel L (2004). Identification of hematopoietic stem/progenitor cells: strength and drawbacks of functional assays. Oncogene.

[60] Schwartz RM, Palsson BO, Emerson SG (1991). Rapid medium perfusion rate significantly increases the productivity and longevity of human bone marrow cultures. Proc. Natl. Acad. Sci. USA..

[61] Koller MR, Emerson SG, Palsson BO (1993). Large-scale expansion of human stem and progenitor cells from bone marrow mononuclear cells in continuous perfusion cultures. Blood.

[62] Sandstrom CE, Bender JG, Miller WM, Papoutsakis ET (1996). Development of novel perfusion chamber to retain nonadherent cells and its use for comparison of human “mobilized” peripheral blood mononuclear cell cultures with and without irradiated bone marrow stroma. Biotechnol. Bioeng..

[63] Berry JD, Godara P, Liovic P, Haylock DN (2015). Predictions for optimal mitigation of paracrine inhibitory signalling in haemopoietic stem cell cultures. Stem Cell Res Ther.

[64] Kapur S, Baylink DJ, Lau KHW (2003). Fluid flow shear stress stimulates human osteoblast proliferation and differentiation through multiple interacting and competing signal transduction pathways. Bone.

[65] Metzger TA, Schwaner SA, LaNeve AJ, Kreipke TC, Niebur GL (2015). Pressure and shear stress in trabecular bone marrow during whole bone loading. J Biomech.

[66] Soves CP (2014). Megakaryocytes are mechanically responsive and influence osteoblast proliferation and differentiation. Bone.

[67] Yourek G, McCormick SM, Mao JJ, Reilly GC (2010). Shear stress induces osteogenic differentiation of human mesenchymal stem cells. Regen Med.

[68] Jiang J, Woulfe DS, Papoutsakis ET (2014). Shear enhances thrombopoiesis and formation of microparticles that induce megakaryocytic differentiation of stem cells. Blood.

[69] Adamo L (2009). Biomechanical forces promote embryonic haematopoiesis. Nature.

[70] Hosseinizand H, Ebrahimi M, Abdekhodaie MJ (2015). Agitation increases expansion of cord blood hematopoietic cells and promotes their differentiation into myeloid lineage. Cytotechnology.

[71] Fan X (2014). Low-dose insulin-like growth factor binding proteins 1 and 2 and angiopoietin-like protein 3 coordinately stimulate *ex vivo* expansion of human umbilical cord blood hematopoietic stem cells as assayed in NOD/SCID gamma null mice. Stem Cell Res Ther.

[72] Zhang CC, Kaba M, Iizuka S, Huynh H, Lodish HF (2008). Angiopoietin-like 5 and IGFBP2 stimulate *ex vivo* expansion of human cord blood hematopoietic stem cells as assayed by NOD/SCID transplantation. Blood.

[73] Huynh H (2008). Insulin-like growth factor-binding protein 2 secreted by a tumorigenic cell line supports *ex vivo* expansion of mouse hematopoietic stem cells. Stem Cells.

[74] Huynh H (2011). IGF binding protein 2 supports the survival and cycling of hematopoietic stem cells. Blood.

[75] Chesney J (1999). An essential role for macrophage migration inhibitory factor (MIF) in angiogenesis and the growth of a murine lymphoma. Mol. Med..

[76] Calandra T, Roger T (2003). Macrophage migration inhibitory factor: a regulator of innate immunity. Nat Rev Immunol.

[77] Baugh JA (2006). Dual regulation of macrophage migration inhibitory factor (MIF) expression in hypoxia by CREB and HIF-1. Biochem. Biophys. Res. Commun..

[78] Raheja LF, Genetos DC, Yellowley CE (2008). Hypoxic osteocytes recruit human MSCs through an OPN/CD44-mediated pathway. Biochem. Biophys. Res. Commun..

[79] Eliasson P, Jonsson JI (2010). The Hematopoietic Stem Cell Niche: Low in Oxygen but a Nice Place to be. J. Cell. Physiol..

[80] Eliasson P (2010). Hypoxia mediates low cell-cycle activity and increases the proportion of long-term reconstituting hematopoietic stem cells during *in vitro* culture. Exp. Hematol..

[81] Ivanovic Z (2013). Respect the anaerobic nature of stem cells to exploit their potential in regenerative medicine. Regen Med.

[82] Csaszar E (2012). Rapid expansion of human hematopoietic stem cells by automated control of inhibitory feedback signaling. Cell Stem Cell.

[83] Muller E (2016). Distinguishing autocrine and paracrine signals in hematopoietic stem cell culture using a biofunctional microcavity platform. Sci Rep.

[84] Vlaski M (2009). Low oxygen concentration as a general physiologic regulator of erythropoiesis beyond the EPO-related downstream tuning and a tool for the optimization of red blood cell production *ex vivo*. Exp. Hematol..

[85] Alepee N (2014). State-of-the-art of 3D cultures (organs-on-a-chip) in safety testing and pathophysiology. ALTEX.

[86] Malet-Martino M, Martino R (2002). Clinical studies of three oral prodrugs of 5-fluorouracil (capecitabine, UFT, S-1): a review. Oncologist.

[87] Cerrato L, Valeri A, Bueren JA, Albella B (2009). *In vitro* sensitivity of granulo-monocytic progenitors as a new toxicological cell system and endpoint in the ACuteTox Project. Toxicol. Appl. Pharmacol..

[88] Pessina A (2003). Application of the CFU-GM assay to predict acute drug-induced neutropenia: an international blind trial to validate a prediction model for the maximum tolerated dose (MTD) of myelosuppressive xenobiotics. Toxicol. Sci..

[89] Pessina A (2001). Prevalidation of a model for predicting acute neutropenia by colony forming unit granulocyte/macrophage (CFU-GM) assay. Toxicol In Vitro.

[90] Cui ZF (2007). Application of multiple parallel perfused microbioreactors and three-dimensional stem cell culture for toxicity testing. Toxicol In Vitro.

[91] Sung JH, Shuler ML (2009). A micro cell culture analog (microCCA) with 3-D hydrogel culture of multiple cell lines to assess metabolism-dependent cytotoxicity of anti-cancer drugs. Lab Chip.

[92] Malerba I, Casati S, Diodovich C, Parent-Massin D, Gribaldo L (2004). Inhibition of CFU-E/BFU-E and CFU-GM colony growth by cyclophosphamide, 5-fluorouracil and taxol: development of a high-throughput *in vitro* method. Toxicol In Vitro.

[93] Rojas-Rios P, Gonzalez-Reyes A (2014). Concise review: The plasticity of stem cell niches: a general property behind tissue homeostasis and repair. Stem Cells.

[94] Trumpp A, Essers M, Wilson A (2010). Awakening dormant haematopoietic stem cells. Nat Rev Immunol.

[95] Bieback K (2009). Human alternatives to fetal bovine serum for the expansion of mesenchymal stromal cells from bone marrow. Stem Cells.

[96] Kleinhans C (2015). A perfusion bioreactor system efficiently generates cell-loaded bone substitute materials for addressing critical size bone defects. Biotechnol J.

[97] Bernad A (1994). Interleukin-6 is required *in vivo* for the regulation of stem cells and committed progenitors of the hematopoietic system. Immunity.

[98] Laterveer L, Lindley IJ, Hamilton MS, Willemze R, Fibbe WE (1995). Interleukin-8 induces rapid mobilization of hematopoietic stem cells with radioprotective capacity and long-term myelolymphoid repopulating ability. Blood.

[99] Fukuda S, Bian H, King AG, Pelus LM (2007). The chemokine GRObeta mobilizes early hematopoietic stem cells characterized by enhanced homing and engraftment. Blood.

[100] Choi YS, Lim DS, Lim SM, Kim DI (2012). Effects of mixed feeder cells on the expansion of CD34(+) cells. J Biosci Bioeng.

[101] Papadopoulou C, Corrigall V, Taylor PR, Poston RN (2008). The role of the chemokines MCP-1, GRO-alpha, IL-8 and their receptors in the adhesion of monocytic cells to human atherosclerotic plaques. Cytokine.

[102] Stetler-Stevenson WG, Bersch N, Golde DW (1992). Tissue inhibitor of metalloproteinase-2 (TIMP-2) has erythroid-potentiating activity. FEBS Lett.

[103] Leng L (2003). MIF signal transduction initiated by binding to CD74. J. Exp. Med..

[104] Stier S (2005). Osteopontin is a hematopoietic stem cell niche component that negatively regulates stem cell pool size. J. Exp. Med..

