# Supplementary material for: 3D models of the hematopoietic stem cell niche under steady-state and active conditions
Source: Sci Rep. 2017 Jul 4;7:4625. doi: 10.1038/s41598-017-04808-0 (PMC5496931; doi:10.1038/s41598-017-04808-0)
Supplement: Supplementary file 1 — Supplementary Figures [file 41598_2017_4808_MOESM1_ESM.pdf]

3D models of the hematopoietic stem cell niche under steady-state and active conditions

Lisa Rödling, Ivo Schwedhelm, Saskia Kraus, Karen Bieback, Jan Hansmann, Cornelia Lee-Thedieck*

SUPPLEMENTARY DATA

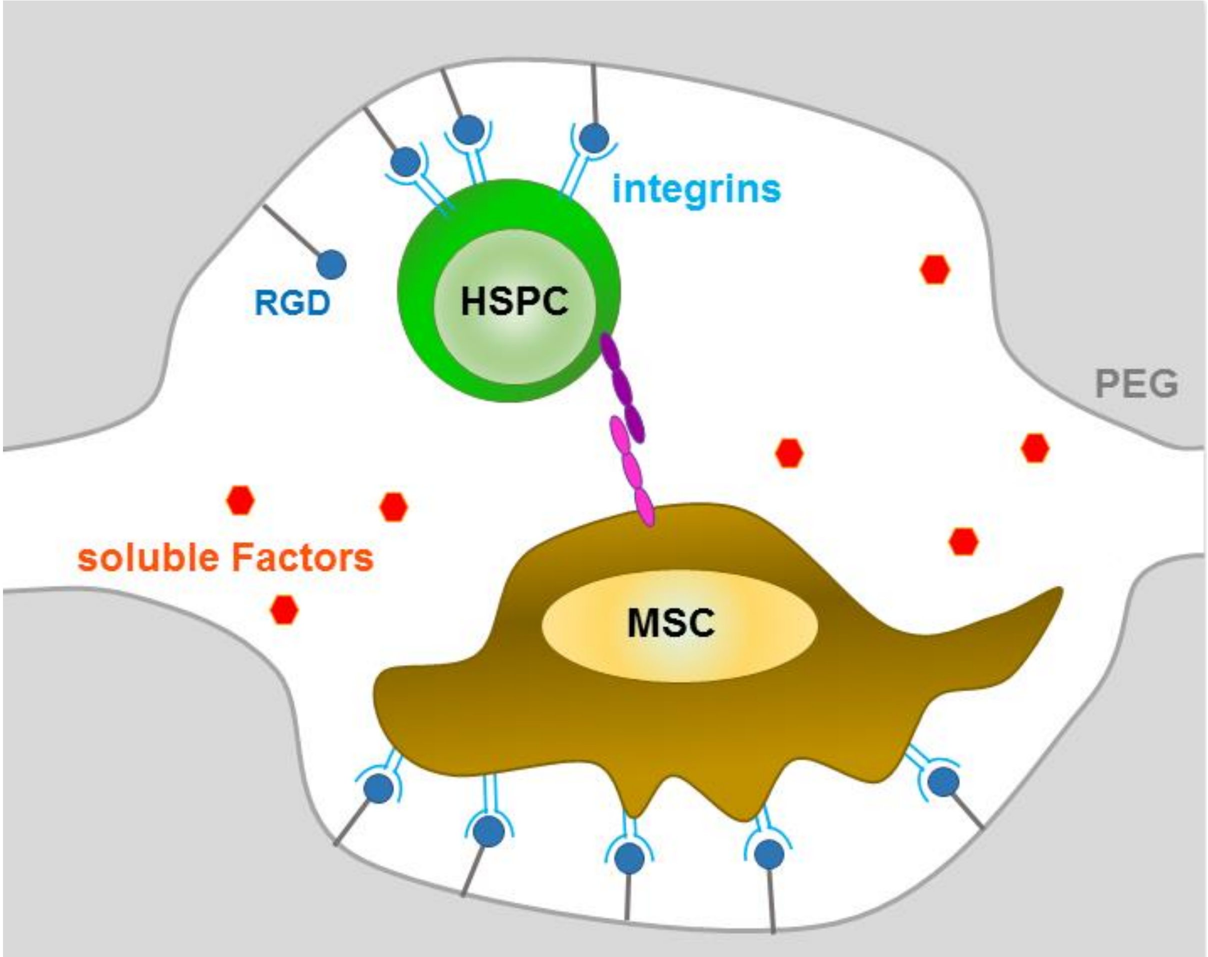

Supplementary Figure S1. Schematic drawing of the applied bone marrow analog. A macroporous PEG hydrogel scaffold (grey) is functionalized with the adhesive peptide RGD (dark blue) by co-polymerization of the peptide into the hydrogel's polymer network. RGD is recognized by the cells via integrin receptors (turquois). The scaffold is seeded with MSCs (brownish) that act as supporting niche cells in co-culture with HSPCs (green), e.g. by direct cell-cell contacts (violet) or secretion of soluble factors (orange).

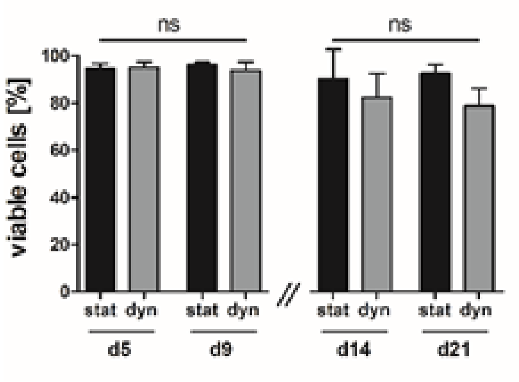

Supplementary Figure S2. Cell viability in 3D scaffolds after static and dynamic culture. Cells were harvested from static and dynamic culture after 5, 9, 14 or 21 days and stained with the dead cell stain *SytoxAADvanced*. The proportion of positively stained (dead) cells was determined by flow cytometric analysis. Stat = static culture, dyn = dynamic culture; the day of analysis is given at the bottom (d5 = day 5, d9 = day 9, day14 = day 14, d21 = day 21); for d5 and d9 N = 4 independent experiments; for d14 and d21 N = 3 independent experiments.
